# Improved prognosis with integrated care management including early rhythm control and healthy lifestyle modification in patients with concurrent atrial fibrillation and diabetes mellitus: a nationwide cohort study

**DOI:** 10.1186/s12933-023-01749-z

**Published:** 2023-01-30

**Authors:** So-Ryoung Lee, Hyo-Jeong Ahn, Eue-Keun Choi, Seung-Woo Lee, Kyung-Do Han, Seil Oh, Gregory Y. H. Lip

**Affiliations:** 1 Department of Internal Medicine, Seoul National University Hospital, 101 Daehak‐ro, Jongno‐gu, 03080 Seoul, Republic of Korea; 2 Department of Internal Medicine, Seoul National University College of Medicine, Seoul, Republic of Korea; 3 Department of Medical Statistics, College of Medicine, Catholic University of Korea, Seoul, Republic of Korea; 4 Statistics and Actuarial Science, Soongsil University, Seoul, Republic of Korea; 5 Liverpool Centre for Cardiovascular Science, University of Liverpool and Liverpool Chest & Heart Hospital, Liverpool, UK; 6 Department of Clinical Medicine, Aalborg University, Aalborg, Denmark

**Keywords:** Early rhythm control, Lifestyle modification, Atrial fibrillation, Diabetes mellitus

## Abstract

**Background:**

Patients with concurrent atrial fibrillation (AF) and diabetes mellitus (DM) [AF-DM] have a high risk of cardiovascular and diabetes-related complications, but are less engaged in a comprehensive treatment approach. We evaluated the association of early rhythm control (ERC), lifestyle modification (LSM), and a combination of ERC and LSM with cardiovascular or diabetes-related complication risk in patients with AF-DM (type 2).

**Methods:**

From the National Health Information Database, 47,940 patients diagnosed with AF-DM in 2009–2016 were included. We defined ERC as rhythm control therapy within two years of AF diagnosis and LSM as adherence to ≥ 2 of the healthy behaviors among non-current smoking, non-drinking, and regular exercise. We compared the primary (ischemic stroke) and secondary (macro- and microvascular complications, glycemic emergency, and all-cause death) outcomes in four groups: non-ERC and non-LSM (group 1), LSM only (group 2), ERC only (group 3), and both ERC and LSM (group 4).

**Results:**

Of total, 10,617 (22%), 26,730 (55.8%), 2,903 (6.1%), and 7,690 (16.0%) were classified into groups 1 to 4, in sequence. The mean duration from AF diagnosis to ERC was 25.6 ± 75.5 days. During 4.0 (interquartile range: 2.5–6.2) years’ follow-up, groups 2 and 3 were associated with 23% and 33% lower risks of stroke than group 1, respectively. Group 4 was associated with the lowest risk of stroke: hazard ratio (HR) 0.58, 95% confidence interval (CI) 0.51–0.67, p < 0.001. Regarding secondary outcomes, the lowest risks were also observed in group 4; macro- and microvascular complications, glycemic emergency, and all-cause death had HRs (95% CIs) of 0.63 (0.56–0.70), 0.88 (0.82–0.94), 0.72 (0.62–0.84), and 0.80 (0.73–0.87), respectively, all p < 0.001.

**Conclusions:**

For AF-DM patients, ERC and LSM exert a synergistic effect in preventing cardiovascular and diabetes-related complications with the greatest lowered risk of stroke. A comprehensive treatment approach should be pursued in AF-DM patients.

**Supplementary Information:**

The online version contains supplementary material available at 10.1186/s12933-023-01749-z.

## Background

Diabetes mellitus (DM) is a common risk factor and often associated with multimorbidity in patients with atrial fibrillation (AF) [1]. Approximately one in four-to-six individuals with AF have DM [2, 3], and approximately 20% of patients with DM have AF [4], with at least two-fold higher prevalence than those without DM [5]. Despite optimal, current guideline-based management, AF remains a major cause of stroke, heart failure, cardiovascular death, and increasing hospitalization and healthcare costs [6]. Furthermore, the coexistence of DM is linked to an even greater risk of cardiovascular events and mortality, particularly a highly significant stroke-risk elevation [7–9]. Therefore, patients with concomitant AF and DM require a holistic or integrated approach to their management, considering the adverse clinical outcomes that influence each other.

Contemporary AF treatment approaches are streamlined into four fundamental pillars: anticoagulation, better symptom care through rhythm and rate control, and cardiovascular risk factor/comorbidity management [10, 11]. Such an approach is now recommended in the guidelines [12, 13], given the improved outcomes and clinical trial data [14, 15].

Among these essential strategies, knowledge of rhythm control and risk-factor management strategies has recently been updated, highlighting the importance of early management based on AF’s progressive nature and an increased cardiovascular-complication risk within the first AF-diagnosis year [16]. The Early Treatment of Atrial Fibrillation for Stroke Prevention Trial (EAST-AFNET 4) trial demonstrated the clinical superiority of early rhythm control over usual care in patients with AF and cardiovascular conditions [17]. In addition, lifestyle modifications, including alcohol abstinence, regular exercise, and smoking cessation around AF diagnosis, are reportedly associated with lower cardiovascular outcomes in patients with AF [18–20].

However, the directionality and magnitude of the effects of these treatment strategies—early rhythm control (ERC), lifestyle modifications (LSM), and a combination of both, primarily in patients with concomitant AF and DM (AF-DM)—have not been evaluated. Specifically, regarding macro- and microvascular complications, glycemic emergency, and all-cause death, the assessment of the clinical effects focused on diabetes-related complications remains unknown. These aspects require attention because patients with AF-DM are reportedly less engaged in rhythm-control intervention and experience greater functional impairment, although they are associated with higher cardiovascular risks [3, 7].

As AF and DM are both chronic conditions exposed to higher cardiovascular complications during their lifetime, a comprehensive approach to care that potentially derives maximal benefit should be pursued [13, 21, 22]. Therefore, we aimed to evaluate the individual beneficial effects of ERC and LSM as well as the synergistic effect of their combination (i.e., both ERC and LSM) on the risk of cardiovascular and diabetes-related complications in patients with AF-DM.

## Methods

The National Health Information Database (NHID; https://nhiss.nhis.or.kr/), which integrates the National Health Insurance Service data of the Republic of Korea, was used to generate a nationwide population-based cohort. The insurance service covers the entire population, and all insured adults are eligible for biennial general health examinations. All insurers’ demographic data, income-based insurance contributions, health examination findings, and medical utilization data (prescriptions, procedures or operation history, and inpatient and outpatient records) are available from the NHID. The NHID also include information on insurers’ death (the date and cause) provided by Statistics Korea [23]. Health examination data included demographic data, anthropometric and laboratory measurements, and self-reported questionnaire responses regarding lifestyle behaviors (smoking status, alcohol consumption, and regular exercise) [23–25]. This study was approved by the Institutional Review Board of Seoul National University Hospital (E-2206-109-1333).

### Study population

**Fig. 1:**
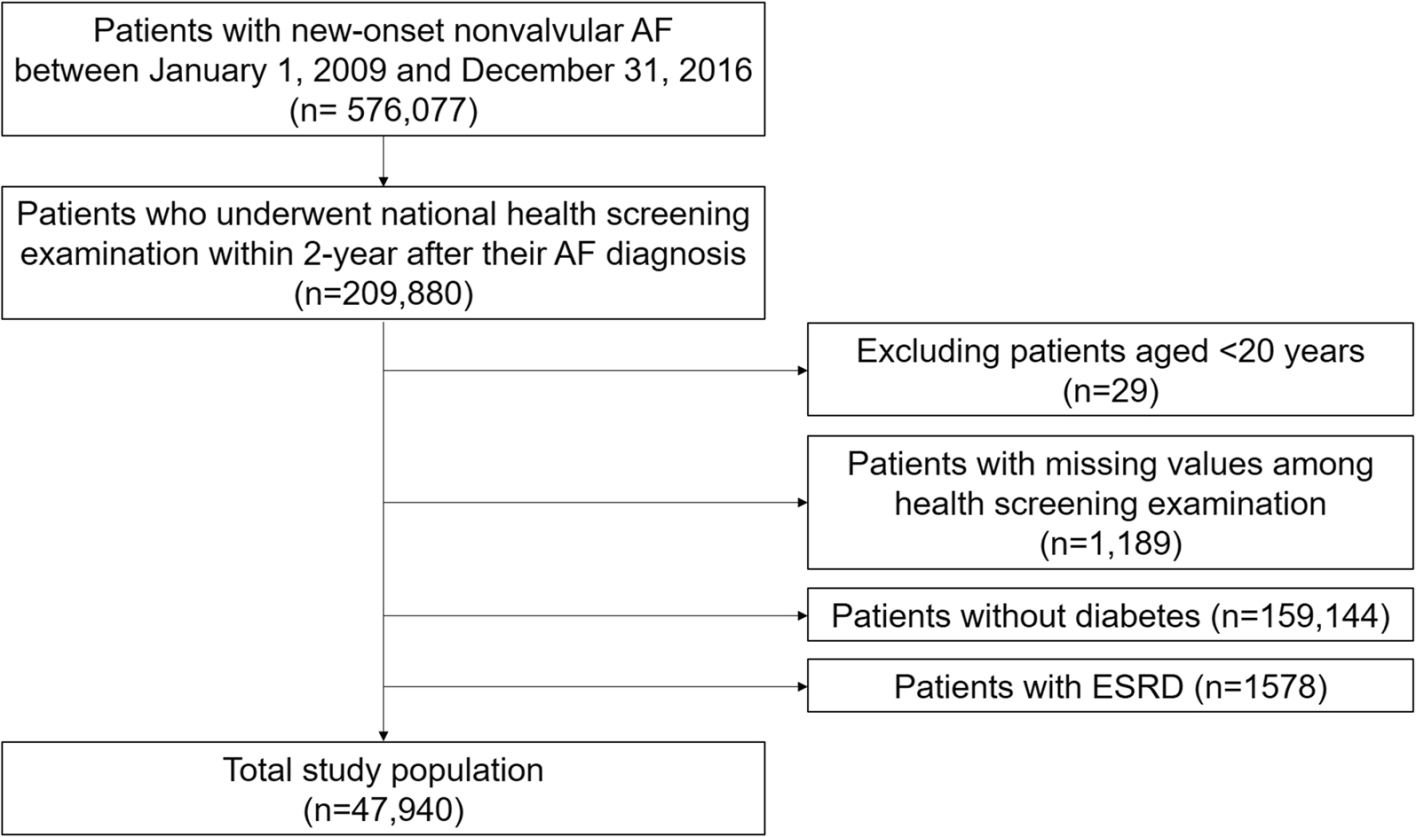
Flow diagram of the study population. *AF*, atrial fibrillation, *ESRD* end-stage renal disease

Patient enrollment is shown in Fig. 1. We initially identified patients with new-onset non-valvular AF between January 1, 2009, and December 31, 2016. Among these, patients with AF who underwent their national health screening examination within 2 years after their AF diagnosis were included (n = 209,880). Patients aged < 20 years, those with missing values in health screening examinations, those without DM (type 2), and those with end-stage renal disease were excluded. Finally, 47,940 patients with concomitant AF and DM (type 2) were included in this study.

### Covariates

We incorporated the diagnostic codes (ICD-10), inpatient and outpatient records, examination results, and prescription records to determine the presence of baseline comorbidities (hypertension, dyslipidemia, heart failure, prior ischemic stroke, prior intracranial hemorrhage, prior myocardial infarction, peripheral artery disease, chronic obstructive pulmonary disease, cancer, chronic liver disease, chronic kidney disease, osteoporosis, hyperthyroidism, hypothyroidism, and sleep apnea). Detailed operational definitions of comorbidities and clinical scores (CHA_2_DS_2_-VASc score and Charlson Comorbidity Index [CCI]) are described in Additional file 1**: **Table S1, S2, and have been validated in previous studies using the NHID cohort [18, 26]. Additional DM data—diabetes duration, classification, and number of prescribed diabetes medications—were assessed as baseline characteristics.

Lifestyle behaviors, which were assessed based on self-reported questionnaire responses during health examinations, were investigated as follows: (1) smoking status (current or not); (2) alcohol consumption (current or non-drinker); and (3) regular exercise (moderate-intensity exercise ≥ 5 times per week, vigorous-intensity exercise ≥ 3 times per week, or lack of regular exercise)[27, 28]. Moderate-intensity exercise was defined as performing ≥ 30 min of brisk-pace walking, playing tennis doubles, or bicycling leisurely; vigorous-intensity exercise was defined as performing ≥ 20 min of running, climbing, fast cycling, or aerobics [19]. Given the absence of a known permissible level of alcohol in patients with AF and a linear incremental risk of clinical outcomes according to the higher alcohol consumption, we discriminate alcohol intake as a dichotomous categorization (current vs. non-drinker) [18, 29]. We defined a healthy lifestyle behavior score (HLS, 0–3) by assigning 1 point each to non-current smokers, non-drinkers, and regular exercisers.

### Early rhythm control, healthy lifestyle, and their combination thereof

Patients were categorized into ERC and non-ERC groups. Those who underwent rhythm-control therapy within 2 years after new-onset AF were assigned to the ERC group. Rhythm-control treatment was defined as any prescription of antiarrhythmic drugs of either class Ic (flecainide, propafenone, or pilsicainide) or class III (amiodarone, sotalol, or dronedarone) as well as direct-current cardioversion or AF catheter ablation. Patients who did not receive any rhythm-control treatment during the screening period were assigned to the non-ERC group.

Regarding lifestyle behaviors, patients were classified into two HLS-based groups. In an HLS-based exploratory analysis of cardiovascular outcomes in patients with AF-DM, we found most patients to be distributed at HLS 1,2, and the number of individuals was statistically comparable between the combination of HLS 0,1 and HLS 2,3. Notably, a stepwise decrease in cardiovascular-outcome risk was observed (Additional file 1: Table S3). Considering the patient distribution and risk–benefit trend, we divided patients into HLS 0,1 and HLS 2,3 groups; HLS 2,3 patients were defined as the healthy lifestyle group and noted as a group implementing LSM only (LSM group).

Considering the aforementioned combined classification of ERC and LSM, we categorized the study population into four groups to evaluate the association between the ERC–LSM combination and clinical-outcome risk as follows: (1) those without ERC and LSM (group 1), (2) those with LSM but without ERC (group 2), (3) those with ERC but without LSM (group 3), and (4) those with both ERC and LSM (group 4).

### Study outcomes and follow-up

The primary outcome was ischemic stroke incidence during the follow-up period. To include overt cerebral ischemia events and clarify the outcome criteria, transient ischemic attack (TIA) was not taken into account as in our previous studies [18, 30]. Four secondary outcomes were evaluated as follows: (1) macrovascular complications, defined as a composite of ischemic stroke, myocardial infarction, and peripheral artery disease; (2) microvascular complications, defined as the composite of retinopathy, neuropathy, and occurrence of end-stage renal disease; (3) glycemic emergency, defined as a composite of diabetes ketoacidosis, hyperosmolar hyperglycemic stage, and hypoglycemia; and (4) all-cause death. Outcomes were defined using diagnostic codes and medical use owing to the relevant clinical events. The operational definitions of the primary and secondary outcomes are presented in Additional file 1: Table S1 [25, 31, 32]. The data of all-cause death was retrieved from the death record of NHID.

The follow-up index date was defined as the baseline health examination date, which was performed within 2 years of AF diagnosis. Patients were followed from the index date to the occurrence of each outcome, death, or end of the study period (December 31, 2018), whichever preceded.

### Statistical analysis

Baseline characteristics are presented as means and standard deviations or medians and interquartile ranges for continuous variables and as numbers and percentages for categorical variables. Continuous and categorical variables were compared between groups using the independent sample t-test and chi-squared test, respectively. Among the four groups categorized by rhythm control and lifestyle, continuous and categorical variables were compared using one-way analysis of variance and the chi-square test to evaluate the significance of the differences, respectively. Study-outcome incidence rates are presented as the number of events per 100 person-years. Hazard ratios (HRs) and the corresponding 95% confidence intervals (CIs) were estimated using Cox proportional hazard regression models.

Crude risks of primary and secondary outcomes were analyzed, and the adjusted results were subsequently evaluated using multivariable Cox regression analyses. Model 1 indicates unadjusted HRs; model 2 was adjusted for age and sex; and model 3 was adjusted for age, sex, CHA_2_DS_2_-VASc score, CCI, diabetes duration, comorbidities (including hypertension, dyslipidemia, heart failure, prior ischemic stroke, myocardial infarction, peripheral artery disease, chronic obstructive pulmonary disease, cancer, chronic liver disease, chronic kidney disease, osteoporosis, hyperthyroidism, hypothyroidism, and sleep apnea), low income, body mass index, systolic blood pressure, fasting glucose, and medications (oral anticoagulation therapy, antiplatelet agents, statins, angiotensin-converting enzyme inhibitor/angiotensin receptor blocker, beta-blocker, non-dihydropyridine calcium channel blocker, dihydropyridine calcium channel blocker, diuretics, digoxin, and type and number of diabetes medications).

Comparison of adjusted cumulative incidence rates among the four groups categorized by early rhythm control/non-rhythm control and healthy/unhealthy lifestyle was based on the adjusted Kaplan–Meier estimates with the log-rank test.

As a sensitivity analysis, a competing risk analysis was performed, considering death as a competing risk based on the Fina and Gray proportional hazards model [33]. All p-values were two-sided, and a p-value < 0.05 was considered statistically significant. All statistical analyses were performed using SAS (version 9.4; SAS Institute, Cary, NC).

## Results

A total of 47,940 patients (mean age: 66.8 ± 10.5 years; 61.9% men) were included in this analysis (**Fig. ****1**). The study population’s baseline characteristics are presented in Table 1. The mean diabetes duration was 5.6 ± 4.7 years (median: 5.0 years; interquartile range, IQR 1.0–9.45 years). Regarding the number of diabetes medications, 35.8%, 26.2%, 20.9%, and 17.2% of patients were prescribed three or more, two, one, and no diabetes medications, respectively. The mean CHA_2_DS_2_-VASc, HAS-BLED, and CCI scores were 4.6 ± 1.9, 3.0 ± 1.3, and 4.5 ± 2.4, respectively. Patients had a high cardiovascular-comorbidity burden; in particular, 92.1% had hypertension, while dyslipidemia was evident in 60.0%, heart failure in 38.1%, prior stroke in 33.0%, and prior myocardial infarction in 15.5%.

**Table 1:** Baseline characteristics of study population

	Total (n = 47,940)	Group 1 (n = 10,617)	Group 2 (n = 26,730)	Group 3 (n = 2903)	Group 4 (n = 7690)	p-value
Rhythm control and Lifestyle						
Duration from AF diagnosis to rhythm control (days)						
Mean ± SD	25.5 ± 75.5	–	–	25.3 ± 74.4	25.6 ± 76.0	0.835
Median (interquartile ranges)	0 (0–8)	–	–	0 (0–8)	0 (0–8)	0.051
≤ 1 year	10,446 (21.79)	–	–	2864 (98.66)	7582 (98.6)	< 0.001
> 1 year	147 (0.31)	–	–	39 (1.34)	108 (1.4)	
Rhythm control treatment						
Antiarrhythmic agents	10,534 (22.0)	0 (0)	0 (0)	2879 (99.2)	7655 (99.5)	< 0.001
Class Ic	5589 (11.7)	0 (0)	0 (0)	1638 (56.4)	3951 (51.4)	< 0.001
Class III	6031 (12.6)	0 (0)	0 (0)	1512 (52.1)	4519 (58.8)	< 0.001
Direct current cardioversion	708 (1.5)	0 (0)	0 (0)	201 (6.9)	507 (6.6)	< 0.001
AF catheter ablation	254 (0.5)	0 (0)	0 (0)	63 (2.2)	191 (2.5)	< 0.001
Unhealthy lifestyle behavior						
Current smoker	7090 (14.8)	5237 (49.3)	412 (1.5)	1332 (45.9)	109 (1.4)	< 0.001
Any drinker	13,859 (28.9)	8718 (82.1)	2071 (7.8)	2420 (83.4)	650 (8.5)	< 0.001
Lack of regular exercise	38,337 (80.0)	9955 (93.8)	20,116 (75.3)	272 2(93.8)	5544 (72.1)	< 0.001
Healthy lifestyle behavior score						
0	3344 (7.0)	2676 (25.2)	0 (0)	668 (23.0)	0 (0)	< 0.001
1	10,176 (21.2)	7941 (74.8)	0 (0)	2235 (77.0)	0 (0)	
2	28,902 (60.3)	0 (0)	22,599 (84.6)	0 (0)	6303 (82.0)	
3	5518 (11.5)	0 (0)	4131 (15.5)	0 (0)	1387 (18.0)	
Age (years), mean ± SD	66.8 ± 10.5	62.0 ± 10.7	69.0 ± 9.9	61.0 ± 10.2	67.8 ± 9.6	< 0.001
< 65	18,922 (39.5)	6230 (58.7)	8168 (30.6)	1847 (63.6)	2677 (34.8)	< 0.001
65 to < 75	17,832 (37.2)	3159 (29.8)	10,733 (40.2)	790 (27.2)	3150 (41.0)	
≥ 75	11,186 (23.3)	1228 (11.6)	7829 (29.3)	266 (9.2)	1863 (24.2)	
Men	29,652 (61.9)	9693(91.3)	13,047 (48.8)	2703 (93.1)	4209 (54.7)	< 0.001
CHA_2_DS_2_-VASc, mean ± SD	4.6 ± 1.9	3.7 ± 1.6	5 ± 1.82	3.6 ± 1.5	4.9 ± 1.8	< 0.001
≥ 3	41,413 (86.4)	7812 (73.6)	24,436 (91.4)	2142 (73.8)	7023 (91.3)	< 0.001
CCI, mean ± SD	4.5 ± 2.4	4.0 ± 2.3	4.7 ± 2.4	3.9 ± 2.2	4.7 ± 2.4	< 0.001
Duration of diabetes (years)						
Mean ± SD	5.6 ± 4.7	4.9 ± 4.5	5.8 ± 4.7	4.9 ± 4.6	5.9 ± 4.8	< 0.001
Median (interquartile ranges)	5.0 (1.0–9.5)	3.9 (0.4–8.7)	5.5 (1.2–9.7)	3.8 (0.6–8.6)	5.5 (1.2–10.1)	< 0.001
Hypertension	44,169 (92.1)	9599 (90.4)	24,707 (92.4)	2660 (91.6)	7203 (93.7)	< 0.001
Dyslipidemia	28,770 (60.0)	5877 (55.4)	16,182 (60.5)	1695 (58.4)	5016 (65.2)	< 0.001
Heart failure	18,259 (38.1)	3176 (29.9)	10,237 (38.3)	1104 (38.0)	3742 (48.7)	< 0.001
Prior ischemic stroke	15,807 (33.0)	2641 (24.9)	10,128 (37.9)	599 (20.6)	2439 (31.7)	< 0.001
Prior ICH	799 (1.7)	113 (1.1)	481 (1.8)	37 (1.3)	168 (2.2)	< 0.001
Prior myocardial infarction	7424 (15.5)	1361 (12.8)	4161 (15.6)	447 (15.4)	1455 (18.9)	< 0.001
Peripheral artery disease	13,314 (27.8)	2718 (25.6)	7811 (29.2)	678 (23.4)	2107 (27.4)	< 0.001
COPD	10,939 (22.8)	1999 (18.8)	6512 (24.4)	549 (18.9)	1879 (24.4)	< 0.001
Cancer	3070 (6.4)	440 (4.1)	2055 (7.7)	87 (3.0)	488 (6.4)	< 0.001
Chronic liver disease	9775 (20.4)	2630 (24.8)	5048 (18.9)	673 (23.2)	1424 (18.5)	< 0.001
Chronic kidney disease	10,946 (22.8)	1513 (14.3)	6933 (25.9)	403 (13.9)	2097 (27.3)	< 0.001
Osteoporosis	9039 (18.9)	819 (7.7)	6493 (24.3)	177 (6.1)	1550 (20.2)	< 0.001
Hyperthyroidism	3944 (8.2)	801 (7.5)	2081 (7.8)	265 (9.1)	797 (10.4)	< 0.001
Hypothyroidism	4558 (9.5)	712 (6.7)	2701 (10.1)	236 (8.1)	909 (11.8)	< 0.001
Sleep apnea	144 (0.3)	36 (0.3)	58 (0.2)	20 (0.7)	30 (0.4)	< 0.001
Low income	9275 (19.4)	2168 (20.4)	5143 (19.2)	576 (19.8)	1388 (18.1)	< 0.001
Health examination						
Body mass index (kg/m2)						
Mean ± SD	25.0 ± 3.6	25.2 ± 3.5	24.9 ± 3.6	25.3 ± 3.4	25.1 ± 3.6	< 0.001
≥ 25	23,190 (48.4)	5307 (50.0)	12,689 (47.5)	1487 (51.2)	3707 (48.2)	< 0.001
Fasting glucose (mg/dL)	134.4 ± 43.8	140.1 ± 45.8	133.1 ± 43.7	137.8 ± 43.6	129.6 ± 40.2	< 0.001
SBP (mmHg)	128.0 ± 16.2	127.6 ± 16.1	128.3 ± 16.3	127.2 ± 15.9	128.0 ± 16.3	< 0.001
Estimated GFR (mL/min)	77.2 ± 31.4	82.7 ± 34.1	75.4 ± 30.5	82.1 ± 30.3	73.7 ± 29.3	< 0.001
Medication						
Oral anticoagulants	23,129 (48.3)	4536 (42.7)	11,993 (44.9)	1702 (58.6)	4898 (63.7)	< 0.001
Warfarin	7066 (14.7)	1374 (12.9)	3796 (14.2)	523 (18.0)	1373 (17.9)	< 0.001
DOAC	16,063 (33.5)	3162 (29.8)	8197 (30.7)	1179 (40.6)	3525 (45.8)	< 0.001
Antiplatelet agent	15,485 (32.3)	3525 (33.2)	8782 (32.9)	915 (31.5)	2263 (29.4)	< 0.001
Aspirin	12,933 (27.0)	3039 (28.6)	7221 (27.0)	797 (27.5)	1876 (24.4)	< 0.001
P2Y12 inhibitor	4869 (10.2)	922 (8.7)	2867 (10.7)	260 (9.0)	820 (10.7)	< 0.001
ACEi/ARB	16,754 (35.0)	3722 (35.1)	9698 (36.3)	910 (31.4)	2424 (31.5)	< 0.001
Beta-blocker	7712 (16.1)	1600 (15.1)	4295 (16.1)	501 (17.3)	1316 (17.1)	< 0.001
Non-DHP CCB	2670 (5.6)	580 (5.5)	1414 (5.3)	182 (6.3)	494 (6.4)	< 0.001
DHP CCB	10,365 (21.6)	2304 (21.7)	6212 (23.2)	494 (17.0)	1355 (17.6)	< 0.001
Diuretics	11,697 (24.4)	2272 (21.4)	7129 (26.7)	536 (18.5)	1760 (22.9)	< 0.001
Digoxin	3828 (8.0)	859 (8.1)	2481 (9.3)	125 (4.3)	363 (4.7)	< 0.001
Statin	12,967 (27.1)	2642 (24.9)	7495 (28.0)	712 (24.5)	2118 (27.5)	< 0.001
Diabetes medication						
Metformin	32,345 (67.5)	6873 (64.74)	18,318 (68.5)	1875 (64.6)	5279 (68.7)	< 0.001
Sulfonylurea	22,708 (47.4)	4795 (45.16)	13,211 (49.4)	1208 (41.6)	3494 (45.4)	< 0.001
Meglitinide	1239 (2.6)	188 (1.77)	787 (2.9)	55 (1.9)	209 (2.7)	< 0.001
Alpha-glucosidase inhibitor	4903 (10.2)	990 (9.32)	2943 (11.0)	224 (7.7)	746 (9.7)	< 0.001
Thiazolidinediones	3460 (7.2)	798 (7.52)	1954 (7.3)	208 (7.2)	500 (6.5)	0.053
DPP4 inhibitors	14,905 (31.1)	3174 (29.9)	8221 (30.8)	907 (31.2)	2603 (33.9)	< 0.001
SGLT-2 inhibitors	487 (1.0)	120 (1.1)	249 (0.9)	40 (1.4)	78 (1.0)	0.070
Glucagon-like peptide-1	16 (0.03)	5 (0.05)	7 (0.03)	0 (0)	4 (0.05)	0.426
Insulin	15,272 (31.9)	2614 (24.6)	9027 (33.8)	761 (26.2)	2870 (37.3)	< 0.001
Number of diabetes medications						
Without any medication	8227 (17.2)	2416 (22.8)	4053 (15.2)	635 (21.9)	1123 (14.6)	< 0.001
1 type	10,035 (20.9)	2003 (18.9)	5660 (21.2)	604 (20.8)	1768 (23.0)	
2 types	12,538 (26.2)	2713 (25.6)	7122 (26.6)	745 (25.7)	1958 (25.5)	
≥ 3 types	17,140 (35.8)	3485 (32.8)	9895 (37.0)	919 (31.7)	2841 (36.9)	

*ACEi* angiotensin-converting enzyme inhibitor, *AF* atrial fibrillation, *ARB* angiotensin receptor blocker, *CCB* calcium channel blocker, *CCI* Charlson Comorbidity Index, *COPD* chronic obstructive pulmonary disease, *DHP* Dihydropyridine, *DOAC*, direct oral anticoagulant, *DPP4* Dipeptidyl peptidase-4, *GFR* glomerular filtration rate, *ICH* intracranial hemorrhage, *SBP* systolic blood pressure, *SD* standard deviation, *SGLT-2* Sodium-glucose cotransporter-2

Of the total population, 22% (n = 10,593) were assigned to the ERC group. Regarding lifestyle, HLS 0, 1, 2, and 3 were found in 7.0%, 21.2%, 60.3%, and 11.5% of total study population, respectively. Based on the definition of a healthy lifestyle (HLS ≥ 2), 71.8% of patients (n = 34,420) were assigned to the LSM group. ERC- and LSM-based baseline characteristics are shown in Additional file 1: Table S4, S5.

The mean duration from AF diagnosis to rhythm control in the ERC group was 25.6 ± 75.5 days (median: 0, IQR 0–8 days), and 98.6% of the ERC group received rhythm control within 1 year after newly diagnosed AF. Among rhythm-control treatments, 99.4% of patients were prescribed anti-arrhythmic drugs, 6.7% underwent DC cardioversion, and 2.4% underwent AF catheter ablation (Additional file 1: Table S4). In the non-LSM group, the prevalence of current smoking, current drinking, and lack of regular physical activity was 48.6%, 82.4%, and 93.8%, respectively.

Regarding the ERC–LSM combination, 10,617 (22%), 26,730 (55.8%), 2903 (6.1%), and 7690 (16.0%) were assigned to groups 1, 2, 3, and 4, respectively. The baseline characteristics according to the classification of group are shown in Table 1.

During a median 4 year follow-up (IQR: 2.5–6.2 years), ischemic stroke occurred in 2779 patients (incidence rate, 1.37 per 100 person-years) in the study population. The crude incidence rates of stroke and secondary outcomes, including macrovascular complications, microvascular complications, hypoglycemic emergency, and mortality, are presented in Table 2 according to ERC, LSM, and their combination.

**Table 2:** Event numbers, crude incidence rates, hazard ratios for the risk of stroke, diabetes-related complications, and death according to the combination of early rhythm control and healthy lifestyle

	Number	Event	IR	Model 1 HR (95% CI)	Model 2 (HR 95% CI)	Model 3 (HR 95% CI)
Primary outcome: Stroke						
Group 1	10,617	622	1.34	1 (reference)	1 (reference)	1 (reference)
Group 2	26,730	1678	1.47	1.098 (1.001–1.204)	0.824 (0.745–0.912)	0.769 (0.694–0.851)
Group 3	2903	114	0.96	0.714 (0.585–0.872)	0.748 (0.613–0.913)	0.670 (0.548–0.819)
Group 4	7690	364	1.18	0.878 (0.771–0.999)	0.697 (0.609–0.797)	0.581 (0.507–0.667)
p-value				< 0.001	< 0.001	< 0.001
Secondary outcomes						
Macrovascular complications						
Group 1	10,617	936	2.06	1 (reference)	1 (reference)	1 (reference)
Group 2	26,730	2420	2.16	1.049 (0.972–1.131)	0.857 (0.790–0.931)	0.794 (0.731–0.863)
Group 3	2903	182	1.56	0.752 (0.642–0.882)	0.783 (0.668–0.917)	0.708 (0.604–0.831)
Group 4	7690	554	1.83	0.883 (0.795–0.981)	0.753 (0.675–0.839)	0.625 (0.559–0.698)
p-value				< 0.001	< 0.001	< 0.001
Microvascular complications						
Group 1	10,617	2195	5.38	1 (reference)	1 (reference)	1 (reference)
Group 2	26,730	6478	6.70	1.225 (1.167–1.286)	1.082 (1.026–1.141)	1.001 (0.949–1.055)
Group 3	2903	465	4.33	0.792 (0.717–0.876)	0.804 (0.727–0.888)	0.830 (0.751–0.918)
Group 4	7690	1547	5.78	1.034 (0.969–1.104)	0.932 (0.871–0.998)	0.875 (0.816–0.937)
p-value				< 0.001	< 0.001	< 0.001
Glycemic emergency						
Group 1	10,617	472	1.00	1 (reference)	1 (reference)	1 (reference)
Group 2	26,730	1439	1.25	1.249 (1.126–1.386)	0.904 (0.807–1.014)	0.802 (0.715–0.900)
Group 3	2903	87	0.73	0.728 (0.579–0.915)	0.762 (0.606–0.957)	0.772 (0.613–0.971)
Group 4	7690	327	1.06	1.061 (0.922–1.222)	0.813 (0.702–0.942)	0.720 (0.620–0.836)
p-value				< 0.001	0.013	< 0.001
All-cause death						
Group 1	10,617	1340	2.79	1 (reference)	1 (reference)	1 (reference)
Group 2	26,730	4443	3.77	1.351 (1.271–1.436)	0.985 (0.923–1.052)	0.896 (0.839–0.957)
Group 3	2903	256	2.11	0.760 (0.665–0.868)	0.825 (0.722–0.944)	0.839 (0.733–0.959)
Group 4	7690	970	3.08	1.109 (1.021–1.204)	0.877 (0.806–0.955)	0.801 (0.734–0.873)
p-value				< 0.001	< 0.001	< 0.001

IR, per 100 person-years

Model 1: unadjusted

Model 2: age and sex adjusted

Model 3: age, sex, CHA_2_DS_2_-VASc score, Charlson comorbidity index, duration of diabetes, hypertension, dyslipidemia, heart failure, prior ischemic stroke, myocardial infarction, peripheral artery disease, chronic obstructive pulmonary disease, cancer, chronic liver disease, chronic kidney disease, osteoporosis, hyperthyroidism, hypothyroidism, sleep apnea, low income, body mass index, systolic blood pressure, fasting glucose, oral anticoagulation therapy, antiplatelet agents, statin, angiotensin-converting enzyme inhibitor/ angiotensin receptor blocker, beta-blocker, non-dihydropyridine calcium channel blocker, dihydropyridine calcium channel blocker, diuretics, digoxin, types and numbers of diabetes medications were adjusted

*CI* confidence interval, *HR* hazard ratio, *IR* incidence rate

### Early rhythm control and the risks of stroke, diabetes-related complications, and mortality

**Fig. 2:**
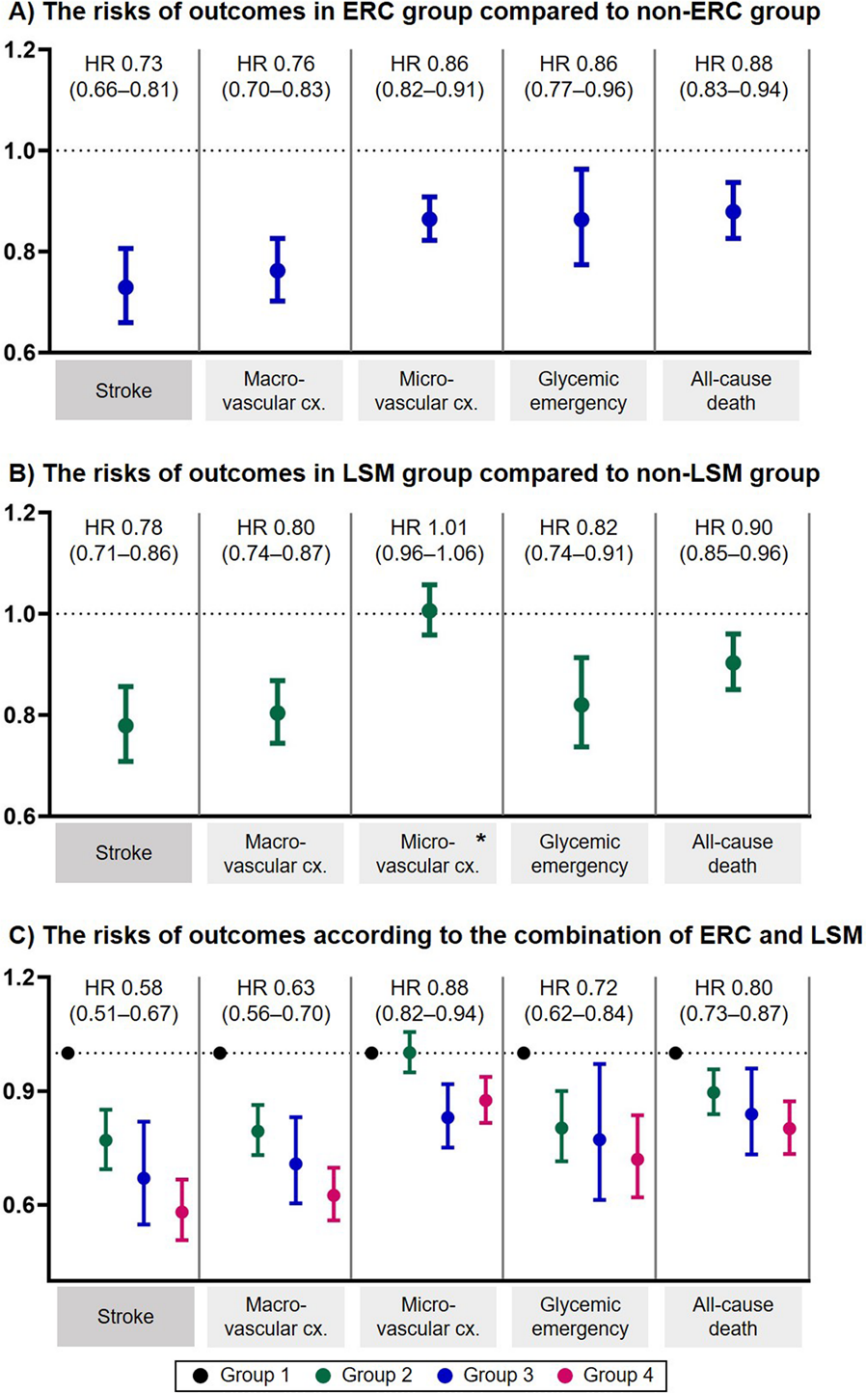
The risks of the primary and secondary outcomes (**A**) in the early rhythm control group compared with those in the non-early rhythm control group, (**B**) in the lifestyle modification group compared with those in the non-lifestyle modification group, and (**C**) according to the combination of early rhythm control and lifestyle modification among patients with concurrent atrial fibrillation and diabetes. All p-values were < 0.05, except for * (p = 0.805). In panel (**C**), the numbered risks are the hazard ratios of group 4 compared with those of group 1. *ERC* early rhythm control, *HR*, hazard ratio, *Cx* complications, *LSM* lifestyle modification

The crude stroke-incidence rates were 1.12 and 1.43 per 100 person-years in the ERC and non-ERC groups, respectively. Regarding the primary outcome, the ERC group was associated with a lower ischemic stroke risk than the non-ERC group (HR 0.729, 95% CI 0.659–0.806, p < 0.001) (Additional file 1: Table S6 and Fig. 2A).

Regarding secondary outcomes, the ERC group exhibited significantly lower hazards of developing macrovascular complications (HR 0.762, 95% CI 0.702–0.826, p < 0.001), microvascular complications (HR 0.864, 95% CI 0.822–0.908, p < 0.001), glycemic emergency (HR 0.863, 95% CI 0.774–0.963, p < 0.001), and all-cause death (HR 0.879, 95% CI 0.826–0.937), p < 0.001) (Additional file 1: Table S6 and Fig. 2A).

### Healthy lifestyle on the risks of stroke, diabetes-related complications, and mortality

The crude stroke-incidence rates were 1.41 and 1.26 per 100 person-years in the LSM and non-LSM groups, respectively. Regarding the primary outcome, the LSM group was associated with a lower stroke risk than the non-LSM group (HR 0.779, 95% CI 0.708–0.856, p < 0.001) (Additional file 1: Table S7 and Fig. 2B).

Regarding the secondary outcomes, lower hazards of macrovascular complications (HR 0.804, 95% CI 0.744–0.868, p < 0.001), glycemic emergency (HR 0.820, 95% CI 0.737–0.913, p < 0.001), and all-cause death (HR 0.903, 95% CI 0.850–0.960, p = 0.001) were observed in the LSM group than in the non-LSM group (Additional file 1: Table S7 and Fig. 2B). The differences in microvascular-complication risks were not statistically significant between the two groups.

### Combination of early rhythm control and a healthy lifestyle on the risks of stroke, macrovascular complications, microvascular complications, glycemic emergency, and mortality

Groups 2 (LSM only) and 3 (ERC only) were associated with lower risks of ischemic stroke (HR, 95% CI 0.769, 0.694–0.851, and 0.670, 0.548–0.819, respectively) than group 1 (non-ERC and non-LSM), with group 4 (both ERC and LSM) being associated with the lowest stroke risk (HR 0.581, 95% CI 0.507–0.667) compared to group 1 (Table 2 and Fig. 2C).

Regarding the secondary outcomes, the lowest risk was also observed in group 4. The HRs (95% CIs) of macrovascular complications, glycemic emergency, and all-cause death were 0.625 (0.559–0.698), 0.720 (0.620–0.836), and 0.801 (0.734–0.873), respectively (all p < 0.001). Regarding microvascular complications, groups 3 (ERC only) and 4 (both ERC and LSM) were associated with statistically lower risks of microvascular complications (HRs 0.830 and 0.875); however, group 2 (LSM only) exhibited a comparable risk to group 1 (HR 1.001 [0.949–1.055]).

**Fig. 3:**
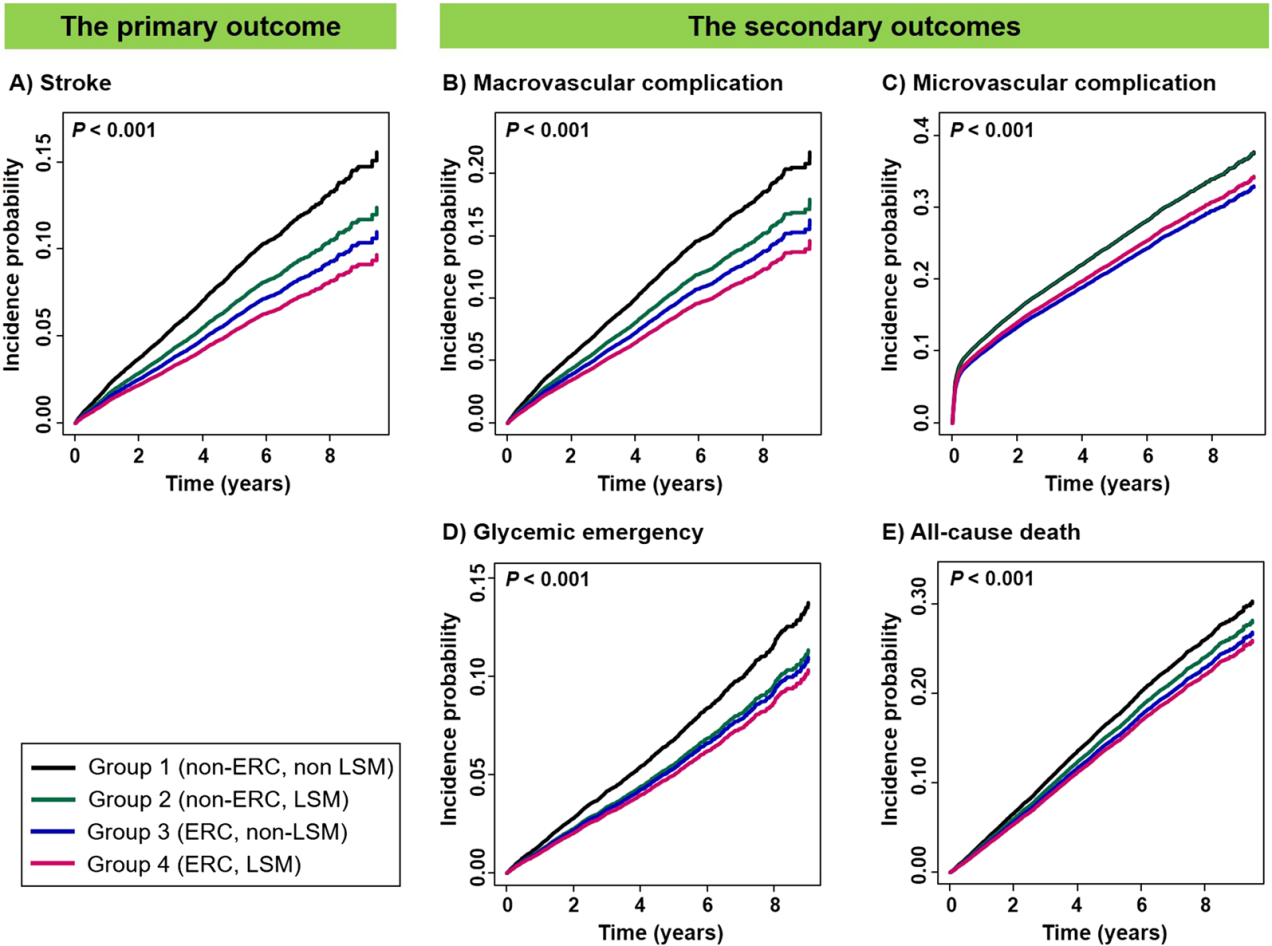
Cumulative risks of the primary and secondary outcomes in patients with concurrent atrial fibrillation and diabetes according to the combination of early rhythm control and a healthy lifestyle: *ERC* early rhythm control; *LSM* lifestyle modification

The cumulative risks of the primary and secondary outcomes according to the ERC–LSM combination are listed in Fig. 3, revealing stepwise risk discrimination with the lowest cumulative risks in group 4.

### Sensitivity analysis: competing risk analysis

Considering death as a competing risk yielded consistent results (Additional file 1: Table S8). The combined ERC–LSM group (group 4) was associated with the lowest risk of stroke compared to group 1 (non-ERC and non-LSM) (HR 0.585, 95% CI 0.511–0.670, p < 0.001).

Group 2 (LSM only) exhibited associations with lower risks of macrovascular complications and glycemic emergencies. In addition to lower risks of macrovascular complications and glycemic emergency, group 3 (ERC only) also had a lower risk of microvascular complications (HR: 0.833, 95% CI 0.753–0.920).

Regarding all secondary outcomes, the lowest risks were observed in group 4 (both ERC and LSM); the HRs (95% CIs) for macro- and microvascular complications and glycemic emergency were 0.627 (0.561–0.701), 0.879 (0.821–0.942), and 0.726 (0.624–0.844), respectively (all p < 0.001).

## Discussion

In this nationwide-cohort study of patients with AF-DM, our major findings were as follows: (1) ERC and LSM were independently associated with 27.1% and 22.1% lower risks of stroke, respectively; (2) both ERC and LSM were linked to lower risks of diabetes-related complications and all-cause mortality, with the greatest decrease in the risk of macrovascular complications of up to 24%; and (3) the ERC–LSM combination had a synergistic risk benefit across all aspects of clinical outcomes: 41.9%, 37.5%, and 19.9% lower risks of stroke, diabetes-related complications, and all-cause death, respectively.

Our results support the individual treatment efficacies of ERC and LSM in patients with DM and newly diagnosed AF and highlight the necessity of simultaneous ERC and LSM approaches as the maximal endeavor to reducing cardiovascular events, diabetes-related complications, and all-cause mortality.

AF is a progressive condition caused by ongoing substrate modification triggered by structural, electrical, and autonomic remodeling. Early AF-management interventions have been suggested to improve AF-related outcomes and reduce mortality [16]. ERC is one of the approaches that interrupt the AF’s pathologic progression, and recent observational studies have confirmed ERC’s clinical benefits over rate control in patients with new-onset AF and cardiovascular conditions [17, 34]. Although one in three-to-four individuals in the EAST-AFNET 4 trial and the emulating nationwide observational cohort study had concomitant AF-DM [17, 34], the two studies included old or highly comorbid patients with AF, with a mean age of approximately 70 years, and almost all were anticoagulated, thus proving potentially unrepresentative of the general AF-DM population.

In the present study, we incorporated a broader spectrum of patients with AF-DM, without limiting younger patients and those who were not mandatorily prescribed oral anticoagulants. We reported a lower stroke risk in the ERC group (HR 0.73) in more heterogeneous patients with AF-DM, and the estimate was comparable to those supported by two prior studies in all patients with AF (HR 0.65–0.74)[17, 34]. Noteworthily, we have first suggested that ERC has favorable outcomes; however, it has also been found to elicit diabetes-specific complications, such as microvascular complications and glycemic emergency, which are preludes for target organ damage and accelerate patients with DM toward adverse outcomes [35, 36]. Unfortunately, patients with AF-DM are pursed less frequently to restore sinus rhythm than patients with AF without DM [3, 7, 8]. Given ERC’s potential universal advantages, this should be advocated as part of the holistic approach to DM-patient management where concurrent AF is present.

Both AF and DM are chronic medical conditions that require lifelong risk-factor modifications as a fundamental means to preventing cardiovascular complications. However, patients with AF generally have an impaired quality of life [37] and those with concomitant DM experience even worse functional ability and poorer quality of life [3, 7, 8]. Additionally, these patients are less mobile, perform less usual activities, and express more diabetes-related psychological discomfort, all of which impede overall well-being [3, 7, 8].

In the present study, we hypothesized that maintaining a healthy lifestyle is independently associated with lower risks of stroke, macrovascular complications, and all-cause death, exhibiting lowered risk estimates comparable to those of ERC. Notably, the HR estimate of glycemic emergency tends to be slightly lower or at least similar in the LSM group compared with that in the ERC group (0.82 vs. 0.86), though superiority was not compared between the LSM and ERC treatment strategies. Engagement in healthy lifestyle behaviors may imply a well-educated status regarding the disease course and importance of self-care and management, resulting in a lower glycemic-emergency rate and better glycemic control. Consistent with the AF and DM management guidelines that emphasize cardiovascular risk-factor modifications [11, 13, 22, 38–40], we suggest that maintaining positive health behaviors potentially achieves key treatment goals.

Another clinical implication of our study is that a more systematic and integrated effort toward managing patients with AF-DM is better and should be preferred to minimize cardiovascular outcomes, diabetes-related complications, and all-cause death. The differentiated synergistic effect of ERC and LSM is consistent with the recent AF and DM guidelines. Indeed, AF and DM treatment requires a comprehensive approach to reduce cardiovascular morbidities, diabetes-related complications, and ultimately, mortality [11, 12, 21, 41]. According to the ERC–LSM combination, a marked decrease was observed in ischemic-stroke risk (HR 0.58 [0.51–0.67]). The augmented favorable effect of the ERC–LSM combination on stroke risk has a significant implication on patients with AF-DM, since the thromboembolic risk, particularly that of stroke, is greatly elevated (79% increase), putting aside other increased risks of morbidity and mortality [9, 42, 43].

The prevalence of AF and DM is expected to rise [13, 44]. The coexistence of both chronic diseases poses more adverse outcomes and a higher financial burden on affected individuals during their lifetime and the society [44–46]. Hence, these comorbid patients require more intensive care, as AF and DM aggravate disease severity. However, they are often associated with less rhythm-control management and poorer quality of life [47]. In this analysis, we comprehensively appraised the clinical impact of current up-to-date treatment strategies in these patients (primarily, early AF-diagnosed patients with DM). Early AF and DM intervention involving both ERC and LSM potentially protects the atrium and prevents systemic reactions against pathologic atrial remodeling, oxidative stress, inflammation, and permanent damage [16, 46, 48]. In response, a holistic or integrated care treatment approach that may mitigate these biological consequences should be pursued, following the contemporary therapeutic goals of AF and DM.

### Limitations

Our study had several limitations. First, it was not possible to obtain information on long-term adherence to rhythm control and the maintenance of sinus restoration. Additionally, data on arrhythmia burden were not available, and its role as a contributor could not be determined. Second, the alteration of treatment strategies—crossover to rhythm control or lifestyle-behavior alteration—during follow-up potentially introduced outcome bias. Third, only Class Ic or Class III antiarrhythmic agent categorization was available; thus, detailed medications in each category could not be classified. Fourth, the individuals were partly on anticoagulation; therefore, residual confounding from the differences in anticoagulation use might have influenced the outcomes, especially stroke, although we adjusted for anticoagulation use during HR estimation. Also, our definition of the primary outcome – ischemic stroke, not including TIA – might underrepresent all the possible cerebral ischemic events. Fifth, our study did not evaluate adverse events related to rhythm control (antiarrhythmic drug use, ablation, or cardioversion). Sixth, we exclusively included patients with early AF who were diagnosed within 2 years; thus, the results may not be generalizable to patients with non-early AF and DM. Seventh, we only included type 2 DM thus the benefit of ERC, LSM, and their synergistic effect in type 1 DM should be evaluated in a more comprehensive cohort. Finally, external generalizability to other ethnic groups should be validated in further studies.

## Conclusion

In patients with concomitant early AF (diagnosed within 2 years) and DM, ERC and LSM were associated with lower risks of stroke, macro- and microvascular complications, glycemic emergency, and all-cause death. The ERC–LSM combination exerts a synergistic effect in preventing cardiovascular and diabetes-related complications, with the greatest lowered risk of stroke. Maximal effort should be invested in developing a comprehensive treatment approach in patients with concurrent AF and DM to reduce stroke, diabetes-related complications, and all-cause death.

## Supplementary Information

**Additional file1** Additional tables (**Table S1~Table S8**).

## Data Availability

The datasets used and analyzed during the current study are available from the corresponding author on reasonable request.
