# Supplementary material for: Improved prognosis with integrated care management including early rhythm control and healthy lifestyle modification in patients with concurrent atrial fibrillation and diabetes mellitus: a nationwide cohort study
Source: Cardiovasc Diabetol. 2023 Jan 30;22:18. doi: 10.1186/s12933-023-01749-z (PMC9887851; doi:10.1186/s12933-023-01749-z)
Supplement: Supplementary file 1 — Additional file1 Additional tables (Table S1~Table S8). [file 12933_2023_1749_MOESM1_ESM.docx]

**Additional file 1**

**Table S1. Definitions of covariates and study outcomes**

**Table S2. Definition of Charlson Comorbidity Index**

**Table S3. Hazard ratios for the risk of stroke, diabetes-related complications, and death according to the healthy lifestyle score**

**Table S4. Baseline characteristics of study groups categorized by early rhythm control**

**Table S5. Baseline characteristics of study groups categorized by lifestyle behavior**

**Table S6. Event numbers, crude incidence rates, hazard ratios for the risk of stroke, diabetes-related complications, and death according to early rhythm control**

**Table S7. Event numbers, crude incidence rates, hazard ratios for the risk of stroke, diabetes-related complications, and death according to lifestyle behavior**

**Table S8. Competing risk analysis for stroke, diabetes-related complications, and death according to the combination of early rhythm control and healthy lifestyle**

**Table S1. Definitions of covariates and study outcomes**

| **Diagnosis** | **ICD-10 code and definition** | **Diagnostic definition** |
| --- | --- | --- |
| **Inclusion/exclusion criteria** |  |  |
| **Atrial fibrillation** | I48.0-48.4, I48.9 | Admission or outpatient department≥1 |
| **Valvular atrial fibrillation** | I05.0, I05.2, I05.9, Z95.2-Z95.4 | Admission or outpatient department≥1 |
| **Diabetes mellitus** | E11-E14 | Admission≥1 or outpatient department≥1 |
|  | Or fasting glucose level ≥ 126 mg/dL | Index health examination |
| **End-stage renal disease** | N18.5, Z49 | Dialysis ≥2  Dialysis: hemodialysis (O7011-O7020), or peritoneal dialysis (O7017, O7075) |
| **Rhythm control strategy** |  |  |
| **Anti-arrhythmic drug** |  |  |
| **Class Ic** | Flecainide, pilsicainide, propafenone |  |
| **Class III** | Amiodarone, sotalol, dronedarone |  |
| **Direct current cardioversion** | Claim codes: M5880 |  |
| **AF catheter ablation** | Claim codes: M6542 or M6547 |  |
| **Comorbidities** |  |  |
| **Hypertension** | I10-I13, I15; and minimum 1 prescription of anti-hypertensive drug (thiazide, loop diuretics, aldosterone antagonist, alpha-/beta-blocker, calcium-channel blocker, angiotensin-converting enzyme inhibitor, angiotensin II receptor blocker). | Admission≥1 or outpatient department≥1 |
|  | Or systolic/diastolic blood pressure ≥ 140/90 mmHg | Index health examination |
| **Dyslipidemia** | E78 | Admission or outpatient department≥1 |
|  | Or Total cholesterol ≥ 240 mg/dL | Index health examination |
| **Heart failure** | I50 | Admission or outpatient department≥1 |
| **Prior ischemic stroke** | I63, I64 | Admission or outpatient department≥1 |
| **Prior intracranial hemorrhage** | I60, I61, I62 | Admission or outpatient department≥1 |
| **Prior myocardial infarction** | I21, I22 | Admission or outpatient department≥1 |
| **Peripheral artery disease** | I70, I73 | Admission or outpatient department≥1 |
| **Chronic obstructive pulmonary disease** | J41-44 | Admission or outpatient department≥1 |
| **Cancer** | C00-97 and RID code (V193) | Admission or outpatient department≥1 |
| **Chronic liver disease** | B18, K70, K71, K72, K73, K74, K76.1 | Admission or outpatient department≥1 |
| **Chronic kidney disease** | Estimated glomerular filtration rate <60 ml/min/1.73m^2^ | Index health examination |
| **Osteoporosis** | M80, M81, M82 (except M82.0) | Admission or outpatient department≥1 |
| **Hyperthyroidism** | E05 | Admission or outpatient department≥1 |
| **Hypothyroidism** | E03 | Admission or outpatient department≥1 |
| **Sleep apnea** | G47.3 | Admission or outpatient department≥1 |
| **Scores** |  |  |
| **CHA_2_DS_2_-VASc score** | Heart failure (1 point), hypertension (1 point), age ≥75 years (2 points), diabetes (1 point), previous stroke/systemic embolism/transient ischemic attack (2 points), vascular disease (prior MI or PAD, 1 point) and female sex (1 point) | |
|  |  |  |
| **Charlson Comorbidity Index** | See Table S 2 |  |
| **Primary outcome** |  |  |
| **Ischemic stroke** | I63, I64 | Primary diagnosis, admission≥1 (≥3 days) and brain imaging (CT or MRI) ≥1 |
| **Secondary outcomes** |  |  |
| **Macrovascular complications** | Composite of ischemic stroke, myocardial infarction, and peripheral artery disease |  |
| **Ischemic stroke** | I63, I64 | Primary diagnosis, admission≥1 (≥3 days) and brain imaging (CT or MRI) ≥1 |
| **Myocardial infarction** | I21, I22 | Primary diagnosis, admission≥1 |
| **Peripheral artery disease** | E08.5, E10.5, E11.5, E13.5, I73.9, I70.2 | Primary diagnosis, admission≥1 |
| **Microvascular complications** | Composite of retinopathy, neuropathy, and end-stage renal disease |  |
| **Retinopathy** | E08.31-E08.35, E08.37, E10.31-E10.35, E10.37, E11.31-E11.35, E11.37, E13.31-E13.35, E13.37 | Admission≥1 or outpatient visits≥3 |
| **Neuropathy** | E08.4, E10.4, E11.4, E13.4 | Admission≥1 or outpatient visits≥3 |
| **End-stage renal disease** | N18.5, Z49 | Dialysis ≥2  Dialysis: hemodialysis (O7011-O7020), or peritoneal dialysis (O7017, O7075) |
| **Glycemic emergency** | Composite of diabetes ketoacidosis, hyperosmolar hyperglycemic state, and hypoglycemia |  |
| **Diabetes ketoacidosis** | E08.1, E10.1, E11.1, E13.1 | Admission or emergency department visit≥1 |
| **Hyperosmolar hyperglycemic state** | E08.0, E11.0, E13.0 | Admission or emergency department visit≥1 |
| **Hypoglycemia** | E16.0-E16.2, E08.64, E10.64, E11.64, E13.64 | Admission or emergency department visit≥1 |

**Table S2. Definition of Charlson Comorbidity Index**

| **Category** | **Weights** | **Disease** | **ICD-10-CM code** |
| --- | --- | --- | --- |
| **Myocardial infarction** | 1 | Acute myocardial infarction | I21 |
|  |  | Subsequent myocardial infarction | I22 |
| **Congestive heart failure** | 1 | Heart Failure | I50 |
| **Peripheral vascular disease** | 1 | Atherosclerosis | I70 |
|  |  | Other peripheral vascular disease | I73 |
| **Cerebrovascular disease** | 1 | Transient cerebral ischemic attacks and related syndromes | G45 |
|  |  | Vascular syndromes of brain in cerebrovascular diseases | G46 |
|  |  | Retinal vascular occlusion | H34 |
|  |  | Cerebrovascular disease | I60-I69 |
| **Dementia** | 1 | Dementia in Alzheimer disease | F00 |
|  |  | Vascular dementia | F01 |
|  |  | Dementia in other disease classified elsewhere | F02 |
|  |  | Unspecified dementia | F03 |
| **Chronic pulmonary disease** | 1 | Chronic lower respiratory diseases | J40-J47 |
|  |  | Lung disease due to external agents | J60-J67 |
| **Rheumatic disease** | 1 | Rheumatoid arthritis with rheumatoid factor | M05 |
| **(connective tissue disorder)** |  | Felty's syndrome | M05.0 |
|  |  | Rheumatoid lung disease with rheumatoid arthritis | M05.1 |
|  |  | Rheumatoid vasculitis with rheumatoid arthritis | M05.2 |
|  |  | Rheumatoid heart disease with rheumatoid arthritis | M05.3 |
|  |  | Rheumatoid myopathy with rheumatoid arthritis | M05.4 |
|  |  | Rheumatoid polyneuropathy with rheumatoid arthritis | M05.5 |
|  |  | Rheumatoid arthritis with involvement of other organs and systems | M05.6 |
|  |  | Rheumatoid arthritis with rheumatoid factor without organ or systems involvement | M05.7 |
|  |  | Other rheumatoid arthritis with rheumatoid factor | M05.8 |
|  |  | Rheumatoid arthritis without rheumatoid factor | M05.9 |
|  |  | Adult-onset Still's disease | M06.1 |
|  |  | Rheumatoid bursitis | M06.2 |
|  |  | Rheumatoid nodule | M06.3 |
|  |  | Inflammatory polyarthropathy | M06.4 |
|  |  | Other specified rheumatoid arthritis | M06.8 |
|  |  | Rheumatoid arthritis, unspecified | M06.9 |
|  |  | Giant cell arteritis with polymyalgia rheumatica | M31.5 |
|  |  | Systemic lupus erythematosus (SLE) | M32 |
|  |  | Drug-induced SLE | M32.0 |
|  |  | SLE with organ or system involvement | M32.1 |
|  |  | Other forms of SLE | M32.8 |
|  |  | SLE, unspecified | M32.9 |
|  |  | Dermatopolymyositis | M33 |
|  |  | Juvenile dermatomyositis | M33.0 |
|  |  | Other dermatomyositis | M33.1 |
|  |  | Polymyositis | M33.2 |
|  |  | Dermatopolymyositis, unspecified | M33.9 |
|  |  | Systemic sclerosis [scleroderma] | M34 |
|  |  | Progressive systemic sclerosis | M34.0 |
|  |  | CR(E)ST syndrome | M34.1 |
|  |  | Systemic sclerosis induced by drug and chemical | M34.2 |
|  |  | Other forms of systemic sclerosis | M34.8 |
|  |  | Systemic sclerosis, unspecified | M34.9 |
|  |  | Other overlap syndromes | M35.1 |
|  |  | Polymyalgia rheumatica | M35.3 |
|  |  | Dermato(poly)myositis in neoplastic disease | M36.0 |
| **Peptic ulcer disease** | 1 | Gastric ulcer | K25 |
|  |  | Duodenal ulcer | K26 |
|  |  | Peptic ulcer, site unspecified | K27 |
|  |  | Gastrojejunal ulcer | K28 |
| **Mild liver disease** | 1 | Chronic viral hepatitis | B18 |
|  |  | Alcoholic fatty liver | K70.0- K70.3, K70.9 |
|  |  | Alcoholic hepatitis |  |
|  |  | Alcoholic fibrosis and sclerosis of liver |  |
|  |  | Alcoholic cirrhosis of liver |  |
|  |  | Alcoholic liver disease, unspecified |  |
|  |  | Toxic liver disease with chronic persistent hepatitis | K71.3- K71.5, K71.7 |
|  |  | Toxic liver disease with chronic lobular hepatitis |  |
|  |  | Toxic liver disease with chronic active hepatitis |  |
|  |  | Toxic liver disease with fibrosis and cirrhosis of liver |  |
|  |  | Chronic hepatitis, not elsewhere classified | K73 |
|  |  | Fibrosis and cirrhosis of liver | K74 |
|  |  | Fatty (change of) liver, not elsewhere classified | K76.0-K76.4, K76.8, K76.9 |
|  |  | Nonalcoholic fatty liver disease |  |
|  |  | Central hemorrhagic necrosis of liver |  |
|  |  | Infarction of liver |  |
|  |  | Hepatic angiomatosis |  |
|  |  | Other specified disease of liver |  |
|  |  | Simple cyst of liver |  |
|  |  | Focal nodular hyperplasia of liver |  |
|  |  | Hepatoptosis |  |
|  |  | Liver disease, unspecified |  |
|  |  | Liver transplant status | Z94.4 |
| **Diabetes without chronic** | 1 | with coma | E10.0, 10.1, 10.6, 10.8, 10.9 |
| **complication** |  | with ketoacidosis | E11.0, 11.1, 11.6, 11.8, 11.9 |
|  |  | with other specified complications | E12.0, 12.1, 12.6, 12.8, 12.9 |
|  |  | with unspecified complications | E13.0, 13.1, 13.6, 13.8, 13.9 |
|  |  | without complications | E14.0, 14.1, 14.6, 14.8, 14.9 |
| **Diabetes with chronic** | 2 | with renal complications | E10.2, 10.3, 10.4, 10.5, 10.7 |
| **complication** |  | with ophthalmic complications | E11.2, 11.3, 11.4, 11.5, 11.7 |
|  |  | with neurologic complications | E12.2, 12.3, 12.4, 12.5, 12.7 |
|  |  | with peripheral circulatory complications | E13.2, 13.3, 13.4, 13.5, 13.7 |
|  |  | with multiple complications | E14.2, 14.3, 14.4, 14.5, 14.7 |
| **Hemi/paraplegia** | 2 | Tropical spastic paraplegia | G04.1 |
|  |  | Hereditary spastic paraplegia | G11.4 |
|  |  | Spastic quadriplegic cerebral palsy | G80.0 |
|  |  | Spastic diplegic cerebral palsy | G80.1 |
|  |  | Spastic hemiplegic cerebral palsy | G80.2 |
|  |  | Flaccid hemiplegia | G81.0 |
|  |  | Spastic hemiplegia | G81.1 |
|  |  | Hemiplegia, unspecified | G81.9 |
|  |  | Flaccid paraplegia | G82.0 |
|  |  | Spastic paraplegia | G82.1 |
|  |  | Paraplegia, unspecified | G82.2 |
|  |  | Flaccid tetraplegia | G82.3 |
|  |  | Spastic tetraplegia | G82.4 |
|  |  | Tetraplegia, unspecified | G82.5 |
|  |  | Diplegia of upper limbs | G83.0 |
|  |  | Paralytic syndrome, unspecified | G83.9 |
| **Renal disease** | 2 | Hypertensive renal disease | I12 |
|  |  | Hypertensive heart and renal disease with renal failure | I13.1 |
|  |  | Chronic nephritic syndrome | N03 |
|  |  | Unspecified nephritic syndrome | N05 |
|  |  | Chronic kidney disease | N18 |
|  |  | Unspecified kidney failure | N19 |
|  |  | Disorders resulting from impaired renal tubular function | N25 |
|  |  | Care involving dialysis | Z49 |
|  |  | Transplanted organ and tissue status - kidney | Z94.0 |
|  |  | Dependence on renal dialysis | Z99.2 |
| **Cancer** | 2 | Any tumor, malignant neoplasm | C00-76, C97 |
|  |  | Any tumor, in situ neoplasm | D00-09 |
|  |  | Any tumor, Benign neoplasm | D10-36 |
|  |  | Any tumor, Neoplasm of unknown behavior | D37-48 |
|  |  | Leukemia | C91-95 |
|  |  | Lymphoma | C81-86 |
| **Metastatic cancer** | 3 | Metastatic solid tumor | C77-80 |
| **Moderate to severe** | 3 | Esophageal varices | I85 |
| **liver disease** |  | Gastric varices | I86.4 |
|  |  | Esophageal varices without bleeding in diseases classified elsewhere | I98.2 |
|  |  | Alcoholic hepatic failure | K70.4 |
|  |  | Toxic liver disease with hepatic necrosis | K71.1 |
|  |  | Hepatic failure (acute/chronic) due to drugs |  |
|  |  | Chronic hepatic failure | K72.1, K72.9 |
|  |  | Hepatic failure, unspecified |  |
|  |  | Hepatic veno-occlusive disease | K76.5-K76.7 |
|  |  | Portal hypertension |  |
|  |  | Hepatorenal syndrome |  |
| **Human immunodeficiency** | 6 | HIV disease resulting in infectious and parasitic diseases | B20 |
| **Virus (HIV)** |  | HIV disease resulting in malignant neoplasm | B21 |
|  |  | HIV disease resulting in other specified diseases | B22 |
|  |  | HIV disease resulting in other conditions | B23 |

**Table S3. Hazard ratios for the risk of stroke, diabetes-related complications, and death according to the healthy lifestyle score**

|  | Number | Event | IR | Model 1  HR (95% CI) | Model 2  HR (95% CI) | Model 3  HR (95% CI) |
| --- | --- | --- | --- | --- | --- | --- |
| **Primary outcome: Stroke** | | | | | | |
| HLS 0 | 3344 | 192 | 1.32 | 1 (reference) | 1 (reference) | 1 (reference) |
| HLS 1 | 10,176 | 544 | 1.24 | 0.935 (0.793-1.102) | 0.734 (0.622-0.867) | 0.7130.604-0.842) |
| HLS 2 | 28,902 | 1768 | 1.47 | 1.104 (0.951-1.281) | 0.675 (0.575-0.791) | 0.613 (0.522-0.720) |
| HLS 3 | 5518 | 274 | 1.13 | 0.854 (0.710-1.027) | 0.576 (0.477-0.696) | 0.525 (0.434-0.635) |
| p-value |  |  |  | <0.001 | <0.001 | <0.001 |
| **Secondary outcomes** | | | | | | |
| **Macrovascular complications** | | | | | | |
| HLS 0 | 3344 | 291 | 2.05 | 1 (reference) | 1 (reference) | 1 (reference) |
| HLS 1 | 10,176 | 827 | 1.92 | 0.936 (0.819-1.070) | 0.766 (0.670-0.877) | 0.751 (0.656-0.860) |
| HLS 2 | 28,902 | 2559 | 2.16 | 1.050 (0.930-1.185) | 0.725 (0.637-0.826) | 0.656 (0.576-0.747) |
| HLS 3 | 5518 | 415 | 1.74 | 0.850 (0.732-0.988) | 0.628 (0.539-0.732) | 0.580 (0.497-0.677) |
| p-value |  |  |  | <0.001 | <0.001 | <0.001 |
| **Microvascular complications** | | | | | | |
| HLS 0 | 3344 | 646 | 5.00 | 1 (reference) | 1 (reference) | 1 (reference) |
| HLS 1 | 10,176 | 2014 | 5.21 | 1.034 (0.946-1.130) | 0.969 (0.886-1.059) | 0.924 (0.845-1.010) |
| HLS 2 | 28,902 | 6739 | 6.54 | 1.274 (1.175-1.381) | 1.061 (0.973-1.156) | 0.943 (0.865-1.028) |
| HLS 3 | 5518 | 1286 | 6.28 | 1.243 (1.131-1.367) | 1.074 (0.975-1.184) | 0.955 (0.866-1.053) |
| p-value |  |  |  | <0.001 | 0.005 | 0.356 |
| **Glycemic emergency** | | | | | | |
| HLS 0 | 3344 | 164 | 1.12 | 1 (reference) | 1 (reference) | 1 (reference) |
| HLS 1 | 10,176 | 395 | 0.89 | 0.792 (0.660-0.950) | 0.634 (0.528-0.761) | 0.612 (0.509-0.735) |
| HLS 2 | 28,902 | 1532 | 1.26 | 1.124 (0.957-1.321) | 0.671 (0.565-0.798) | 0.573 (0.481-0.683) |
| HLS 3 | 5518 | 234 | 0.96 | 0.856 (0.701-1.045) | 0.568 (0.462-0.697) | 0.501 (0.407-0.616) |
| p-value |  |  |  | <0.001 | <0.001 | <0.001 |
| **All-cause death** | | | | | | |
| HLS 0 | 3344 | 399 | 2.67 | 1 (reference) | 1 (reference) | 1 (reference) |
| HLS 1 | 10,176 | 1197 | 2.65 | 0.993 (0.887-1.112) | 0.691 (0.616-0.774) | 0.719 (0.642-0.807) |
| HLS 2 | 28,902 | 4810 | 3.86 | 1.449 (1.308-1.604) | 0.784 (0.704-0.872) | 0.726 (0.652-0.809) |
| HLS 3 | 5518 | 603 | 2.43 | 0.909 (0.801-1.031) | 0.556 (0.489-0.633) | 0.552 (0.485-0.628) |
| p-value |  |  |  | <0.001 | <0.001 | <0.001 |

IR, per 100 person-years

Model 1: unadjusted

Model 2: age and sex adjusted

Model 3: age, sex, CHA_2_DS_2_-VASc score, Charlson Comorbidity Index, duration of diabetes, hypertension, dyslipidemia, heart failure, prior ischemic stroke, myocardial infarction, peripheral artery disease, chronic obstructive pulmonary disease, cancer, chronic liver disease, chronic kidney disease, osteoporosis, hyperthyroidism, hypothyroidism, sleep apnea, low income, body mass index, systolic blood pressure, fasting glucose, oral anticoagulation therapy, antiplatelet agents, statin, angiotensin-converting enzyme inhibitor/ angiotensin receptor blocker, beta-blocker, non-dihydropyridine calcium channel blocker, dihydropyridine calcium channel blocker, diuretics, digoxin, types and numbers of diabetes medications adjusted

Abbreviation: CI, confidence interval; HLS, healthy lifestyle score; HR, hazard ratio; IR, incidence rate.

**Table S4. Baseline characteristics of study groups categorized by early rhythm control**

|  | Non-rhythm control group  (n=37,347) | Early rhythm control group  (n=10,593) | p-value |
| --- | --- | --- | --- |
| Duration from AF diagnosis to rhythm control (days) |  |  |  |
| Mean ± SD | - | 25.5±75.5 | n/a |
| Median (interquartile ranges) | - | 0 (0-8) | n/a |
| ≤ 1 year |  | 10446 (98.61) | n/a |
| > 1 year |  | 147 (1.39) | n/a |
| Rhythm control treatment |  |  |  |
| Antiarrhythmic agents | 0 (0) | 10,534 (99.4) | <0.001 |
| Class Ic | 0 (0) | 5589 (52.8) | <0.001 |
| Class III | 0 (0) | 6031 (56.9) | <0.001 |
| Direct current cardioversion | 0 (0) | 708 (6.7) | <0.001 |
| AF catheter ablation | 0 (0) | 254 (2.4) | <0.001 |
| Unhealthy lifestyle behavior |  |  |  |
| Current smoker | 5649 (15.1) | 1441 (13.6) | <0.001 |
| Any drinker | 10,789 (28.9) | 3070 (29.0) | 0.852 |
| Lack of regular exercise | 30,071 (80.5) | 8266 (78.0) | <0.001 |
| Healthy lifestyle behavior score |  |  | <0.001 |
| 0 | 2676 (7.2) | 668 (6.3) |  |
| 1 | 7941 (21.3) | 2235 (21.1) |  |
| 2 | 22,599 (60.5) | 6303 (59.5) |  |
| 3 | 4131 (11.1) | 1387 (13.2) |  |
| Age (years), mean ± SD | 67.0±10.6 | 65.9±10.2 | <0.001 |
| <65 | 14,398 (38.6) | 4524 (42.7) | <0.001 |
| 65 to <75 | 13,89 2(37.2) | 3940 (37.2) |  |
| ≥75 | 9057 (24.3) | 2129 (20.1) |  |
| Men | 22,740 (60.9) | 6912(65.3) | <0.001 |
| CHA_2_DS_2_-VASc, mean ± SD | 4.6±1.9 | 4.5±1.8 | <0.001 |
| CCI, mean ± SD | 4.5±2.3 | 4.5±2.4 | 0.036 |
| Duration of diabetes (years) |  |  |  |
| Mean ± SD | 5.6±4.7 | 5.7±4.8 | 0.053 |
| Median (interquartile ranges) | 5.0 (1.0-9.4) | 5.0 (1.1-9.7) | 0.951 |
| Hypertension | 34,306 (91.9) | 9863 (93.1) | <0.001 |
| Dyslipidemia | 22,059 (59.1) | 6711 (63.4) | <0.001 |
| Heart failure | 13,413 (35.9) | 4846 (45.8) | <0.001 |
| Prior ischemic stroke | 12,769 (34.2) | 3038 (28.7) | <0.001 |
| Prior ICH | 594 (1.6) | 205 (1.9) | 0.014 |
| Prior myocardial infarction | 5522 (14.8) | 1902 (18.0) | <0.001 |
| Peripheral artery disease | 10,529 (28.2) | 2785 (26.3) | <0.001 |
| COPD | 8511(22.8) | 2428 (22.9) | 0.775 |
| Cancer | 2495 (6.7) | 575 (5.43) | <0.001 |
| Chronic liver disease | 7678 (20.6) | 2097 (19.8) | 0.085 |
| Chronic kidney disease | 8446 (22.6) | 2500 (23.6) | 0.032 |
| Osteoporosis | 7312 (19.6) | 1727 (16.3) | <0.001 |
| Hyperthyroidism | 2882 (7.7) | 1062 (10.0) | <0.001 |
| Hypothyroidism | 3413 (9.1) | 1145 (10.8) | <0.001 |
| Sleep apnea | 94 (0.3) | 50 (0.5) | <0.001 |
| Low income | 7311 (19.6) | 1964 (18.5) | 0.017 |
| Health examination |  |  |  |
| Body mass index (kg/m2) |  |  |  |
| Mean ± SD | 25.0±3.6 | 25.1±3.5 | 0.005 |
| ≥25 | 17,996 (48.2) | 5194 (49.0) | 0.123 |
| Fasting glucose (mg/dL) | 135.1±44.4 | 131.8±41.3 | <0.001 |
| SBP (mmHg) | 128.1±16.2 | 127.8±16.2 | 0.092 |
| Estimated GFR (mL/min) | 77.5±31.8 | 76.0±29.8 | <0.001 |
| Medication |  |  |  |
| Oral anticoagulants | 16,529 (44.3) | 6600 (62.3) | <0.001 |
| Warfarin | 5170 (13.8) | 1896 (17.9) | <0.001 |
| DOAC | 11,359(30.4) | 4704 (44.4) | <0.001 |
| Antiplatelet agent | 12,307 (33.0) | 3178 (30) | <0.001 |
| Aspirin | 10,260 (27.5) | 2673(25.2) | <0.001 |
| P2Y12 inhibitor | 3789 (10.2) | 1080 (10.2) | 0.880 |
| ACEi/ARB | 13,420 (35.9) | 3334 (31.5) | <0.001 |
| Beta-blocker | 5895 (15.8) | 1817 (17.2) | <0.001 |
| Non-DHP CCB | 1994 (5.3) | 676 (6.4) | <0.001 |
| DHP CCB | 8516 (22.8) | 1849 (17.5) | <0.001 |
| Diuretics | 9401 (25.2) | 2296 (21.7) | <0.001 |
| Digoxin | 3340 (8.9) | 488 (4.6) | <0.001 |
| Statin | 10,137 (27.1) | 2830 (26.7) | 0.382 |
| Diabetes medication |  |  |  |
| Metformin | 25,191 (67.5) | 7154 (67.5) | 0.870 |
| Sulfonylurea | 18,006 (48.2) | 4702 (44.4) | <0.001 |
| Meglitinide | 975 (2.6) | 264 (2.5) | 0.497 |
| Alpha-glucosidase inhibitor | 3933 (10.5) | 970 (9.3) | <0.001 |
| Thiazolidinediones | 2752 (7.4) | 708 (6.7) | 0.016 |
| DPP4 inhibitors | 11,395 (30.5) | 3510 (33.1) | <0.001 |
| SGLT-2 inhibitors | 369 (1.0) | 118 (1.1) | 0.254 |
| Glucagon-like peptide-1 | 12 (0.03) | 4 (0.04) | 0.779 |
| Insulin | 11,641 (31.2) | 3631 (34.3) | <0.001 |
| Number of diabetes medications |  |  | <0.001 |
| Without any medication | 6469 (17.3) | 1758 (16.6) |  |
| 1 type | 7663 (20.5) | 2372 (22.4) |  |
| 2 types | 9835 (26.3) | 2703 (25.5) |  |
| ≥3 types | 13,380 (35.8) | 3760 (35.5) |  |

Abbreviation: ACEi, angiotensin-converting enzyme inhibitor; AF, atrial fibrillation; ARB, angiotensin receptor blocker; CCB, calcium channel blocker; CCI, Charlson Comorbidity Index; COPD, chronic obstructive pulmonary disease; DHP, Dihydropyridine; DOAC, direct oral anticoagulant; DPP4, Dipeptidyl peptidase-4, GFR, glomerular filtration rate; ICH, intracranial hemorrhage; SBP, systolic blood pressure; SD, standard deviation; SGLT-2, Sodium-glucose cotransporter-2.

**Table S5. Baseline characteristics of study groups categorized by lifestyle behavior**

|  | Unhealthy lifestyle group  (n=13,520) | Healthy lifestyle group  (n=34,420) | p-value |
| --- | --- | --- | --- |
| Duration from AF diagnosis to rhythm control (days) |  |  |  |
| Mean ± SD | 25.3±74.4 | 25.6±76.0 | 0.835 |
| Median (interquartile ranges) | 0 (0-8) | 0 (0-8) | 0.051 |
| Rhythm control treatment |  |  |  |
| Antiarrhythmic agents | 2879 (21.3) | 7655 (22.2) | 0.024 |
| Class Ic | 1638 (12.1) | 3951 (11.5) | 0.050 |
| Class III | 1512 (11.2) | 4519 (13.1) | <0.001 |
| Direct current cardioversion | 201 (1.5) | 507 (1.5) | 0.910 |
| AF catheter ablation | 63 (0.5) | 191 (0.6) | 0.227 |
| Unhealthy lifestyle behavior |  |  |  |
| Current smoker | 6569 (48.6) | 521 (1.5) | <0.001 |
| Any drinker | 11,138 (82.4) | 2721 (7.9) | <0.001 |
| Lack of regular exercise | 12,677 (93.8) | 25,660 (74.6) | <0.001 |
| Healthy lifestyle behavior score |  |  | <0.001 |
| 0 | 3344 (24.7) | 0 (0) |  |
| 1 | 10,176 (75.3) | 0 (0) |  |
| 2 | 0 (0) | 28,902 (84.0) |  |
| 3 | 0 (0) | 5518 (16.0) |  |
| Age (years), mean ± SD | 61.8±10.6 | 68.8±9.8 | <0.001 |
| <65 | 8077 (59.7) | 10,845 (31.5) | <0.001 |
| 65 to <75 | 3949 (29.2) | 13,883 (40.3) |  |
| ≥75 | 1494 (11.1) | 9692 (28.2) |  |
| Men | 12,396 (91.7) | 17,256 (50.1) | <0.001 |
| CHA_2_DS_2_-VASc, mean ± SD | 3.7±1.6 | 4.97±1.81 | <0.001 |
| CCI, mean ± SD | 4.0±2.3 | 4.7±2.4 | <0.001 |
| Duration of diabetes (years) |  |  |  |
| Mean ± SD | 4.9±4.5 | 5.8±4.7 | <0.001 |
| Median (interquartile ranges) | 3.9 (0.5-8.7) | 5.5 (1.2-9.8) | <0.001 |
| Hypertension | 12,259 (90.7) | 31,910 (92.7) | <0.001 |
| Dyslipidemia | 7572 (56.0) | 21,198 (61.6) | <0.001 |
| Heart failure | 4280 (31.7) | 13,979 (40.6) | <0.001 |
| Prior ischemic stroke | 3240 (24.0) | 12,567 (36.5) | <0.001 |
| Prior ICH | 150 (1.1) | 649 (1.9) | <0.001 |
| Prior myocardial infarction | 1808 (13.4) | 5616 (16.3) | <0.001 |
| Peripheral artery disease | 3396 (25.1) | 9918 (28.8) | <0.001 |
| COPD | 2548 (18.9) | 8391 (24.4) | <0.001 |
| Cancer | 527 (3.9) | 2543 (7.4) | <0.001 |
| Chronic liver disease | 3303 (24.4) | 6472 (18.8) | <0.001 |
| Chronic kidney disease | 1916 (14.2) | 9030 (26.2) | <0.001 |
| Osteoporosis | 996 (7.4) | 8043 (23.4) | <0.001 |
| Hyperthyroidism | 1066 (7.9) | 2878 (8.4) | 0.087 |
| Hypothyroidism | 948 (7.0) | 3610 (10.5) | <0.001 |
| Sleep apnea | 56 (0.4) | 88 (0.3) | 0.004 |
| Low income | 2744 (20.3) | 6531(19.0) | 0.001 |
| Health examination |  |  |  |
| Body mass index (kg/m2) |  |  |  |
| Mean ± SD | 25.2±3.5 | 25.0±3.6 | <0.001 |
| ≥25 | 6794 (50.3) | 16,396 (47.6) | <0.001 |
| Fasting glucose (mg/dL) | 139.6±45.3 | 132.3±43.0 | <0.001 |
| SBP (mmHg) | 127.5±16.0 | 128.2±16.3 | <0.001 |
| Estimated GFR (mL/min) | 82.6±33.4 | 75.1±30.3 | <0.001 |
| Medication |  |  |  |
| Oral anticoagulants | 6238 (46.1) | 16,891 (49.1) | <0.001 |
| Warfarin | 1897 (14.0) | 5169 (15.0) | 0.006 |
| DOAC | 4341 (32.1) | 11,722 (34.1) | <0.001 |
| Antiplatelet agent | 4440 (32.8) | 11,045(32.1) | 0.113 |
| Aspirin | 3836 (28.4) | 9097 (26.4) | <0.001 |
| P2Y12 inhibitor | 1182 (8.7) | 3687 (10.7) | <0.001 |
| ACEi/ARB | 4632 (34.3) | 12,122 (35.2) | 0.047 |
| Beta-blocker | 2101 (15.5) | 5611 (16.3) | 0.041 |
| Non-DHP CCB | 762 (5.6) | 1908 (5.5) | 0.690 |
| DHP CCB | 2798 (20.7) | 7567 (22.0) | 0.002 |
| Diuretics | 2808 (20.8) | 8889 (25.8) | <0.001 |
| Digoxin | 984 (7.3) | 2844 (8.3) | <0.001 |
| Statin | 3354 (24.8) | 9613 (27.9) | <0.001 |
| Diabetes medication |  |  |  |
| Metformin | 8748 (64.7) | 23,597 (68.6) | <0.001 |
| Sulfonylurea | 6003 (44.4) | 16,705 (48.5) | <0.001 |
| Meglitinide | 243 (1.8) | 996 (2.9) | <0.001 |
| Alpha-glucosidase inhibitor | 1214 (9.0) | 3689 (10.7) | <0.001 |
| Thiazolidinediones | 1006 (7.4) | 2454 (7.1) | 0.236 |
| DPP4 inhibitors | 4081 (30.2) | 10,824 (31.5) | 0.007 |
| SGLT-2 inhibitors | 160 (1.2) | 327 (1.0) | 0.021 |
| Glucagon-like peptide-1 | 5 (0.04) | 11 (0.03) | 0.786 |
| Insulin | 3375 (25.0) | 11,897 (34.6) | <0.001 |
| Number of diabetes medications |  |  | <0.001 |
| Without any medication | 3051 (22.6) | 5176 (15.0) |  |
| 1 type | 2607 (19.3) | 7428 (21.6) |  |
| 2 types | 3458 (25.6) | 9080 (26.4) |  |
| ≥3 types | 4404 (32.6) | 12,736 (37.0) |  |

Abbreviation: ACEi, angiotensin-converting enzyme inhibitor; AF, atrial fibrillation; ARB, angiotensin receptor blocker; CCB, calcium channel blocker; CCI, Charlson Comorbidity Index; COPD, chronic obstructive pulmonary disease; DHP, Dihydropyridine; DOAC, direct oral anticoagulant; DPP4, Dipeptidyl peptidase-4, GFR, glomerular filtration rate; ICH, intracranial hemorrhage; SBP, systolic blood pressure; SD, standard deviation; SGLT-2, Sodium-glucose cotransporter-2.

**Table S6. Event numbers, crude incidence rates, hazard ratios for the risk of stroke, diabetes-related complications, and death according to early rhythm control**

|  | Number | Event | IR | Model 1  HR (95% CI) | Model 2  HR (95% CI) | Model 3  HR (95% CI) |
| --- | --- | --- | --- | --- | --- | --- |
| **Primary outcome: Stroke** | | | | | | |
| Non-rhythm control | 37,347 | 2300 | 1.43 | 1 (reference) | 1 (reference) | 1 (reference) |
| Early rhythm control | 10,593 | 478 | 1.12 | 0.778 (0.705-0.859) | 0.815 (0.739-0.900) | 0.729 (0.659-0.806) |
| p-value |  |  |  | <0.001 | <0.001 | <0.001 |
| **Secondary outcomes** | | | | | | |
| **Macrovascular complications** | | | | | | |
| Non-rhythm control | 37,347 | 3356 | 2.13 | 1 (reference) | 1 (reference) | 1 (reference) |
| Early rhythm control | 10,593 | 736 | 1.75 | 0.819 (0.756-0.887) | 0.848 (0.783-0.919) | 0.762 (0.702-0.826) |
| p-value |  |  |  | <0.001 | <0.001 | <0.001 |
| **Microvascular complications** | | | | | | |
| Non-rhythm control | 37,347 | 8673 | 6.31 | 1 (reference) | 1 (reference) | 1 (reference) |
| Early rhythm control | 10,593 | 2012 | 5.36 | 0.833 (0.794-0.875) | 0.850 (0.8009-0.892) | 0.864 (0.822-0.908) |
| p-value |  |  |  | <0.001 | <0.001 | <0.001 |
| **Glycemic emergency** | | | | | | |
| Non-rhythm control | 37,347 | 1911 | 1.18 | 1 (reference) | 1 (reference) | 1 (reference) |
| Early rhythm control | 10,593 | 414 | 0.97 | 0.823 (0.740-0.915) | 0.864 (0.777-0.961) | 0.863 (0.774-0.963) |
| p-value |  |  |  | <0.001 | 0.007 | 0.008 |
| **All-cause death** | | | | | | |
| Non-rhythm control | 37,347 | 5783 | 3.49 | 1 (reference) | 1 (reference) | 1 (reference) |
| Early rhythm control | 10,593 | 1226 | 2.81 | 0.810 (0.761-0.861) | 0.876 (0.823-0.932) | 0.879 (0.826-0.937) |
| p-value |  |  |  | <0.001 | <0.001 | <0.001 |

IR, per 100 person-years; Adjusted models are described in the Table S 2.

Abbreviation: CI, confidence interval; HR, hazard ratio; IR, incidence rate.

**Table S7. Event numbers, crude incidence rates, hazard ratios for the risk of stroke, diabetes-related complications, and death according to lifestyle behavior**

|  | Number | Event | IR | Model 1  HR (95% CI) | Model 2  HR (95% CI) | Model 3  HR (95% CI) |
| --- | --- | --- | --- | --- | --- | --- |
| **Primary outcome: Stroke** | | | | | | |
| Unhealthy lifestyle (HLS 0,1) | 13,520 | 736 | 1.26 | 1 (reference) | 1 (reference) | 1 (reference) |
| Healthy lifestyle (HLS 2,3) | 34,420 | 2042 | 1.41 | 1.117 (1.026-1.215) | 0.836 (0.761-0.918) | 0.779 (0.708-0.856) |
| p-value |  |  |  | 0.010 | <0.001 | <0.001 |
| **Secondary outcomes** | | | | | | |
| **Macrovascular complications** | | | | | | |
| Unhealthy lifestyle (HLS 0,1) | 13,520 | 1118 | 1.95 | 1 (reference) | 1 (reference) | 1 (reference) |
| Healthy lifestyle (HLS 2,3) | 34,420 | 2974 | 2.09 | 1.068 (0.997-1.144) | 0.870 (0.806-0.939) | 0.804 (0.744-0.868) |
| p-value |  |  |  | 0.061 | <0.001 | <0.001 |
| **Microvascular complications** | | | | | | |
| Unhealthy lifestyle (HLS 0,1) | 13,520 | 2660 | 5.16 | 1 (reference) | 1 (reference) | 1 (reference) |
| Healthy lifestyle (HLS 2,3) | 34,420 | 8025 | 6.50 | 1.237 (1.184-1.293) | 1.090 (1.038-1.144) | 1.006 (0.958-1.057) |
| p-value |  |  |  | <0.001 | <0.001 | 0.805 |
| **Glycemic emergency** | | | | | | |
| Unhealthy lifestyle (HLS 0,1) | 13,520 | 559 | 0.95 | 1 (reference) | 1 (reference) | 1 (reference) |
| Healthy lifestyle (HLS 2,3) | 34,420 | 1766 | 1.21 | 1.280 (1.164-1.408) | 0.926 (0.833-1.030) | 0.820 (0.737-0.913) |
| p-value |  |  |  | <0.001 | 0.155 | <0.001 |
| **All-cause death** | | | | | | |
| Unhealthy lifestyle (HLS 0,1) | 13,520 | 1596 | 2.65 | 1 (reference) | 1 (reference) | 1 (reference) |
| Healthy lifestyle (HLS 2,3) | 34,420 | 5413 | 3.62 | 1.366 (1.292-1.444) | 0.994 (0.936-1.056) | 0.903 (0.850-0.960) |
| p-value |  |  |  | <0.001 | 0.841 | 0.001 |

IR, per 100 person-years; Adjusted models are described in the Table S 2.

Abbreviation: CI, confidence interval; HLS, healthy lifestyle; HR, hazard ratio; IR, incidence rate.

**Table S8. Competing risk analysis for stroke, diabetes-related complications, and death according to the combination of early rhythm control and healthy lifestyle**

|  | Number | Event | IR | Model 1  HR (95% CI) | Model 2  HR (95% CI) | Model 3  HR (95% CI) |
| --- | --- | --- | --- | --- | --- | --- |
| **Primary outcome: Stroke** | | | | | | |
| Group 1 | 10617 | 622 | 13.40 | 1 (reference) | 1 (reference) | 1 (reference) |
| Group 2 | 26730 | 1678 | 14.73 | 1.098 (1.001-1.204) | 0.824 (0.745-0.912) | 0.772 (0.696-0.856) |
| Group 3 | 2903 | 114 | 9.61 | 0.714 (0.585-0.872) | 0.748 (0.613-0.913) | 0.667 (0.545-0.816) |
| Group 4 | 7690 | 364 | 11.85 | 0.878 (0.771-0.999) | 0.697 (0.609-0.797) | 0.585 (0.511-0.670) |
| p-value |  |  |  | <0.001 | <0.001 | <0.001 |
| **Secondary outcomes** | | | | | | |
| **Macrovascular complications** | | | | | | |
| Group 1 | 10617 | 1637 | 37.75 | 1 (reference) | 1 (reference) | 1 (reference) |
| Group 2 | 26730 | 4175 | 39.28 | 1.037 (0.979-1.098) | 0.938 (0.881-0.998) | 0.797 (0.732-0.867) |
| Group 3 | 2903 | 395 | 35.72 | 0.933 (0.836-1.041) | 0.958 (0.858-1.069) | 0.708 (0.604-0.831) |
| Group 4 | 7690 | 1180 | 41.74 | 1.085 (1.007-1.170) | 1.003 (0.928-1.083) | 0.627 (0.561-0.701) |
| p-value |  |  |  | 0.034 | 0.088 | <0.001 |
| **Microvascular complications** | | | | | | |
| Group 1 | 10617 | 2195 | 53.76 | 1 (reference) | 1 (reference) | 1 (reference) |
| Group 2 | 26730 | 6478 | 67.02 | 1.225 (1.167-1.286) | 1.082 (1.026-1.141) | 1.006 (0.954-1.061) |
| Group 3 | 2903 | 465 | 43.28 | 0.792 (0.717-0.876) | 0.804 (0.727-0.888) | 0.833 (0.753-0.920) |
| Group 4 | 7690 | 1547 | 57.76 | 1.034 (0.969-1.104) | 0.932 (0.871-0.998) | 0.879 (0.821-0.942) |
| p-value |  |  |  | <0.001 | <0.001 | <0.001 |
| **Glycemic emergency** | | | | | | |
| Group 1 | 10617 | 472 | 10.03 | 1 (reference) | 1 (reference) | 1 (reference) |
| Group 2 | 26730 | 1439 | 12.53 | 1.249 (1.126-1.386) | 0.904 (0.807-1.014) | 0.811 (0.723-0.910) |
| Group 3 | 2903 | 87 | 7.28 | 0.728 (0.579-0.915) | 0.762 (0.606-0.957) | 0.777 (0.618-0.978) |
| Group 4 | 7690 | 327 | 10.61 | 1.061 (0.922-1.222) | 0.813 (0.702-0.942) | 0.726 (0.624-0.844) |
| p-value |  |  |  | <0.001 | 0.014 | <0.001 |

IR, per 100 person-years

Model 1: unadjusted

Model 2: age and sex adjusted

Model 3: age, sex, CHA_2_DS_2_-VASc score, Charlson Comorbidity Index, duration of diabetes, hypertension, dyslipidemia, heart failure, prior ischemic stroke, myocardial infarction, peripheral artery disease, chronic obstructive pulmonary disease, cancer, chronic liver disease, chronic kidney disease, osteoporosis, hyperthyroidism, hypothyroidism, sleep apnea, low income, body mass index, systolic blood pressure, fasting glucose, oral anticoagulation therapy, antiplatelet agents, statin, angiotensin-converting enzyme inhibitor/ angiotensin receptor blocker, beta-blocker, non-dihydropyridine calcium channel blocker, dihydropyridine calcium channel blocker, diuretics, digoxin, types and numbers of diabetes medications adjusted

Abbreviation: CI, confidence interval; HLS, healthy lifestyle score; HR, hazard ratio; IR, incidence rate.
